# A computational tool to detect DNA alterations tailored to formalin-fixed paraffin-embedded samples in cancer clinical sequencing

**DOI:** 10.1186/s13073-018-0547-0

**Published:** 2018-06-07

**Authors:** Mamoru Kato, Hiromi Nakamura, Momoko Nagai, Takashi Kubo, Asmaa Elzawahry, Yasushi Totoki, Yuko Tanabe, Eisaku Furukawa, Joe Miyamoto, Hiromi Sakamoto, Shingo Matsumoto, Kuniko Sunami, Yasuhito Arai, Yutaka Suzuki, Teruhiko Yoshida, Katsuya Tsuchihara, Kenji Tamura, Noboru Yamamoto, Hitoshi Ichikawa, Takashi Kohno, Tatsuhiro Shibata

**Affiliations:** 1 Department of Bioinformatics, National Cancer Center Research Institute, Chuo-ku, Tokyo, 104-0045 Japan; 2 Division of Cancer Genomics, National Cancer Center Research Institute, Chuo-ku, Tokyo, 104-0045 Japan; 3 Department of Clinical Genomics, National Cancer Center Research Institute, Chuo-ku, Tokyo, 104-0045 Japan; 4 Department of Experimental Therapeutics, National Cancer Center Hospital, Chuo-ku, Tokyo, 104-0045 Japan; 5 Division of Genetics, National Cancer Center Research Institute, Chuo-ku, Tokyo, 104-0045 Japan; 6 Division of Translational Genomics, Exploratory Oncology Research & Clinical Trial Center, National Cancer Center, Kashiwa, Chiba, 277-8577 Japan; 7 Division of Genome Biology, National Cancer Center Research Institute, Chuo-ku, Tokyo, 104-0045 Japan; 8 Department of Computational Biology and Medical Sciences, Graduate School of Frontier Sciences, The University of Tokyo, Kashiwa-shi, Chiba, 277-8568 Japan; 9 Laboratory of Molecular Medicine, Human Genome Center, The Institute of Medical Science, The University of Tokyo, Minato-ku, Tokyo, 108-8639 Japan

## Abstract

Advanced cancer genomics technologies are now being employed in clinical sequencing, where next-generation sequencers are used to simultaneously identify multiple types of DNA alterations for prescription of molecularly targeted drugs. However, no computational tool is available to accurately detect DNA alterations in formalin-fixed paraffin-embedded (FFPE) samples commonly used in hospitals. Here, we developed a computational tool tailored to the detection of single nucleotide variations, indels, fusions, and copy number alterations in FFPE samples. Elaborated multilayer noise filters reduced the inherent noise while maintaining high sensitivity, as evaluated in tumor-unmatched normal samples using orthogonal technologies. This tool, cisCall, should facilitate clinical sequencing in everyday diagnostics. It is available at https://www.ciscall.org.

**Electronic supplementary material:**

The online version of this article (10.1186/s13073-018-0547-0) contains supplementary material, which is available to authorized users.

## Background

In recent years, large-scale cancer genome projects such as the International Cancer Genome Consortium [1–3] (ICGC) and The Cancer Genome Atlas (TCGA) have greatly expanded the available knowledge on genomic alterations in cancer. Along with this increasing knowledge, the number of investigational and approved drugs that target aberrant gene products continues to grow [4]. Genomics technologies that have matured through research are now being translated to the clinical setting. In cancer clinical sequencing, next-generation sequencing (NGS) is applied to identify genetic alterations in biopsy or surgical specimens [4–6]. The detected variants are used as targets for molecularly targeted drugs. The advantage of NGS technologies is that they allow the simultaneous detection of various types of aberrations, i.e., single nucleotide variations (SNVs), indels, copy number alterations (CNAs), and gene fusions, in a multitude of genes.

A practical application of clinical sequencing is the identification of DNA alterations in the exons of hundreds of genes in formalin-fixed paraffin-embedded (FFPE) samples, as reported by Frampton et al. [6]. FFPE samples are the first choice for clinical sequencing because such archival samples are needed for mandatory pathological examination, and their storage at room temperature is substantially less costly than that of fresh frozen tissues. One critical issue is the accurate calling of DNA alterations from FFPE-based sequencing data. Chemical processing damages and fragments genomic DNA, resulting in increased error rates and artificial base substitution bias [6–8]. Moreover, low tumor purity [6] and the non-availability of matched normal samples and panels of normal (PON) samples [9] are frequent problems peculiar to clinical sequencing that arise owing to practical and ethical reasons.

Most current computational tools [9–21] for calling cancer DNA alterations have been developed for exploratory research, mostly assuming the use of fresh frozen samples with relatively high tumor purity for Illumina exome/genome sequencing. Some tools for SNVs assume low tumor content but high read depth [22, 23]. Clearly, these tools are not optimal for FFPE sequencing. One successful variant caller for FPPE samples has been reported by a private company [6]; however, the software is not publicly available.

Here, we report the development of an accurate caller termed “**c**l**i**nical **s**equencing **call**er” (cisCall), specialized for identifying DNA alterations from FFPE samples. cisCall is composed of cisMuton, cisFusion, and cisCton, which respectively call SNVs/indels, DNA gene fusions, and CNAs. We show that this computational tool exhibits high performance under a variety of experimental conditions. In this report, we focus on the bioinformatics research aspects of the present calling tool for FFPE samples. The regulatory or clinical testing standards, as well as the clinical significance and the validity of experimental processes (which have been discussed elsewhere [24]), are beyond the scope of this work.

## Methods

### Materials

Sequencing data were derived from cell lines (HCC78 and NCI-H2228), patient samples, and a commercial sample. HCC78 and NCI-H2228 were provided by Dr. John D. Minna of the UT Southwestern Medical Center. Snap-frozen tumor and normal tissues as well as FFPE archival samples that had been obtained at diagnosis were provided by the National Cancer Center (NCC) Biobank. A commercial synthetic human FFPE sample, HD200, was purchased from Horizon (Cambridge, United Kingdom). Twenty normal DNA samples were extracted from noncancerous lung tissues deposited in the NCC Biobank (the biobank did not collect control non-pathological FFPE samples). Half of the lung tissues were from smokers. From the mixture of the 20 normal DNA samples, an unmatched pooled sample was prepared and used as a background dataset in alteration calling. In total, 70 FFPE clinical samples were used as foreground datasets for SNV/indel analysis, and 75 FFPE clinical samples were used as foreground datasets for CNA analysis (five samples were increased because CNA analysis was performed later than SNV analysis). The details on samples are summarized in Additional file 1: Table S1. We validated alterations in 27 and 23 FFPE samples for SNV/indel and CNA analyses, respectively.

Genomic DNA from FFPE tissues was prepared with a QIAamp DNA FFPE tissue kit (Qiagen, Hilden, Germany) and quantified using a Qubit dsDNA BR assay kit (Thermo Fisher Scientific, Waltham, MA, USA) as well as quantitative PCR analysis. The ratio of PCR-amplifiable DNA to total dsDNA indicates DNA quality. When this quality value was ≥ 0.1, samples were retained for sequencing. We further selected FFPE samples with a pathologically measured tumor purity of ≥ 10%.

### Targeted Illumina sequencing

We used custom gene panels for target capture sequencing: the NCC oncopanel v1 (all exons of 134 tumor-related genes and introns of three fusion genes) and v2 (all exons of 90 tumor-related genes and introns of 35 fusion genes; Additional file 2: Table S2) and the NCC Hospital East oncopanel (all exons of 121 tumor-related genes and introns of 12 fusion genes). The bait libraries were designed with SureDesign (Agilent Technologies, Santa Clara, CA, USA). Sequencing libraries were prepared using SureSelect XT reagent (Agilent Technologies), and paired-end read sequencing was performed on MiSeq or HiSeq sequencers (Illumina, San Diego, CA, USA).

### Targeted Ion sequencing

We used custom gene panels for target capture sequencing: the NCC oncopanel v1 and the RET panel (37 fusion genes), the latter of which was specifically designed for genes fused with RET [25–28]. The bait libraries were designed with SureDesign, and the sequencing libraries were prepared using SureSelect XT reagent. For amplicon sequencing, we used the commercial Ion AmpliSeq Cancer Hotspot Panel v2 (hotspot regions of 50 genes; Thermo Fisher Scientific). The sequencing libraries were prepared using an Ion AmpliSeq Library Kit (Thermo Fisher). Single-end read sequencing was performed on Ion PGM or Proton sequencers (Thermo Fisher).

### Validation of SNVs/indels by mass spectrometry

SNVs/indels were validated by iPLEX SNP genotyping using Sequenom MassARRAY, according to the manufacturer’s instructions (Agena Bioscience, San Diego, CA, USA). PCR primers and an extended primer were designed using Assay Design Suite software (Agena Bioscience). After PCR amplification and single-nucleotide extension, data were collected on the MassARRAY Analyzer 4 system.

### Validation of CNAs by qPCR

CNAs were validated by qPCR using TaqMan Fast Universal Master Mix and TaqMan probes (Additional file 3: Table S3) on an Applied Biosystems 7500 Fast Sequence Detection System according to the manufacturer’s instructions (Applied Biosystems, Foster City, CA, USA). Samples were run in triplicate and standardized against endogenous RNase P with RNase P Detection Reagents Kit (Applied Biosystems).

### cisMuton

cisMuton was developed for variation calling from Illumina sequencing data from FFPE samples and Illumina/Ion sequencing data from frozen tissues. cisMuton uses FASTQ and BAM file formats. The algorithm comprises prep filters, the variant extraction step, and eight and nine noise filters for Illumina and Ion sequencing data, respectively (Additional file 2: Figure S1). The prep filters filter out reads based on mapping and base qualities. The variant extraction step uses several statistics derived from Fisher’s exact test to detect A/C/G/T and indels at each chromosomal position. The subsequent noise filter consists of three sets. The first set contains the misalignment filter, strand-bias filter, and others, for which we utilized as many statistical tests and internal controls as possible. Filters in the second set remove erroneous reads and trim erroneous read ends, followed by a second Fisher’s exact test for the remaining reads. Filters in the third set remove errors that escaped the previous filters, utilizing statistical tests based on variant allele frequencies (VAFs). The algorithmic details are described in Additional file 2: Text S1.

### cisFusion

cisFusion is a fusion caller applicable to single-end (Ion) and paired-end (Illumina) DNA sequence reads. cisFusion searches for a gene fusion of which at least one gene is indicated by a user. The algorithm consists of the “2map” and “VF” steps for the single-end mode, and further of the “paired-end” step for the paired-end mode (Additional file 2: Figure S2). The 2map step searches for reads that are mapped to two different genes on the right and left ends, with fusion breakpoints. The VF step saves reads that are missed by the 2map step because of too short alignment, using “virtual fusion” sequences constructed from reads found in the 2map step. The paired-end step searches for R1 and R2 reads between which a fusion breakpoint exists. The algorithmic details are described in Additional file 2: Text S1.

### cisCton

cisCton first executes a GC-content correction, in which locally weighted scatterplot smoothing (LOWESS) regression between binned depths and GC content is performed to correct the depths. Then, it performs circular binary segmentation (CBS) with a non-parametric statistic (the Mann–Whitney *U* statistic) for log*R* calculated from the GC-corrected depths. For a fast computation, cisCton splits a chromosome into windows of a specified size, within which it performs CBS to finally compile the CBS results to chromosome-size segments. It then executes the abortion process: it aborts a segment if the number of individual log*R* values that deviate from the median log*R* of a segment exceeds a threshold. Finally, cisCton defines amplifications or deletions by a bootstrapping approach. The algorithmic details are described in Additional file 2: Text S1.

### Performance evaluation

Details of the procedures for performance evaluation are presented in Additional file 2: Text S1.

## Results

We evaluated cisCall using maximally 75 FFPE samples from a clinical study for entry into early-phase clinical trials at the National Cancer Center Japan [24]. In the study, all exons of 90 genes and reportedly translocated introns of 35 fusion genes (12 kinases and 23 partners) were captured by our original gene panel (NCC oncopanel v2; Additional file 2: Table S2). These exons and introns were sequenced for the detection of SNVs/indels, CNAs, and DNA gene fusions. Target capturing and sequencing were performed using Agilent SureSelect and Illumina MiSeq. Paired-end 150-base sequencing reads were obtained. Reads from a tumor sample and from a frozen sample mixed with noncancerous samples of 20 individuals were used as test (foreground) and control (background) datasets to call DNA alterations, respectively.

The FFPE samples were mostly from breast (31%), gastric (29%), and ovarian (14%) cancers; the histologically determined median tumor content was 40% (interquartile range of 25–65%). The median sequencing depth was 760× (interquartile range, 526–903×). The traceable storage time of all but one FFPE sample sequenced on the basis of the threshold of the FFPE sample quality (Methods) was less than 10 years. For more detailed information about the samples, please refer to Additional file 1: Table S1 and Additional file 2: Figure S3.

### SNV/indel calling

#### Features of cisMuton

cisMuton detects SNVs/indels from targeted sequencing data. It extracts variants using a non-parametric test, Fisher’s exact test [10], by statistically comparing the numbers of A/C/G/T and insertions/deletions of a tumor sample with those of a control sample at each chromosomal position. We chose this method because a non-parametric test makes fewer assumptions than model-based (likelihood or Bayesian) methods; no assumptions are made on Phred scores or error rates, for which the calibration and degrees may differ between FFPE and frozen samples and between different experimental conditions.

cisMuton is characterized by elaborate noise filters to manage the high level of noise in FFPE samples (Additional file 2: Figure S1). Variant extraction methods such as the frequency cutoff method [29] and likelihood or Bayesian methods [11–15, 22, 23] alone do not suffice to filter out noise that arises from errors correlated between different chromosomal positions, such as misalignment. Because we observed that FFPE samples produce many correlated errors, we focused on the improvement of noise filters by incorporating multiple, robust statistical tests and internal controls (e.g., error rates calculated from the data), resulting in a greater flexibility to handle data with different qualities.

We devised the following filters: 1) misalignment filter, 2) strand-bias filter, 3) within-long-homopolymer filter, 4) MQ0 filter, 5) read-end-call filter, 6) surrounded-by-dust filter, 7) abnormal-BQ-drop filter (for Ion-derived indels only), 8) second Fisher filter combined with mismatch filter and trim filter, and 9) VAF-lees filter.

For example, the VAF-lees filter removes “lees” of calls that show suspiciously low VAFs but are not filtered by the other filters for unknown reasons. The distribution of VAFs is regarded as a beta-mixture distribution and the component beta distributions are automatically detected by the expectation maximization algorithm [30]. The algorithm calculates the ICL-BIC criterion [31] to select the best model of all models with different numbers (ranging from 1 to 10) of beta components. The algorithm then searches for the beta component for which the distribution’s average is within a range of low frequencies (e.g., 1–3%), which means that a peak of the VAFs is found at such a low value. It regards such a component as an error distribution, and performs a beta-binomial test for variant and depth counts in a tumor sample to remove lees. cisMuton itself does not have any hard cut-off for VAFs, though variants with low VAFs may be removed from a clinical viewpoint by the tumor board. The algorithmic details and illustrations of all filters are presented in Additional file 2: Text S1 and Additional file 2: Figure S1, respectively. The execution time of cisMuton is typically 1 h 50 min ± 22 (standard deviation (s.d.)) min on a 10-core 2.0 GHz CPU with 264 GB memory for FASTQ files with 7.9 ± 0.6 million reads (either R1 or R2 reads) with 150-bp length.

#### Performance evaluation of cisMuton

**Fig. 1:**
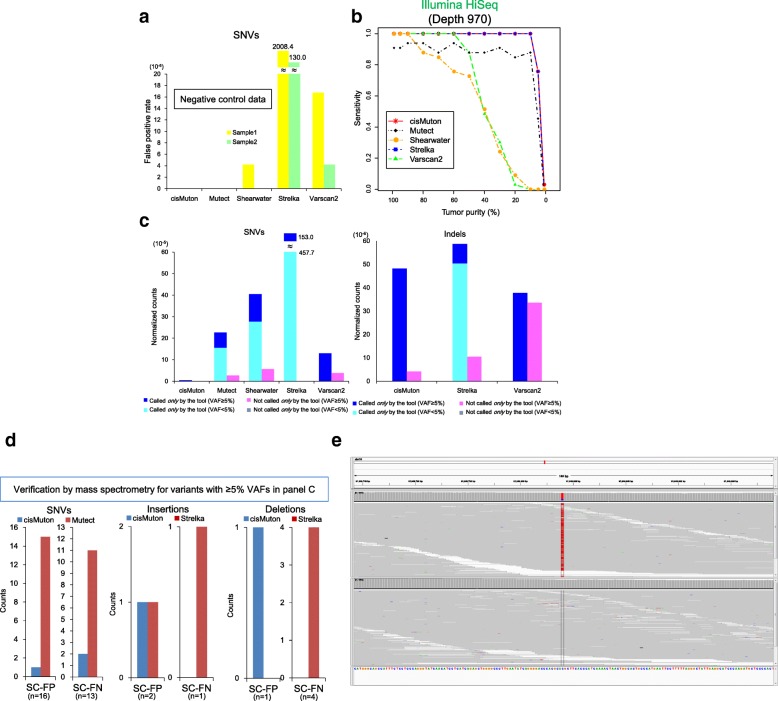
cisMuton calls. **a** False-positive SNV calls in negative control data where tumor FFPE (for foreground data) and tumor frozen samples (for background data) were taken from the same tissue block. The numbers were normalized by target region size (477 k bp). **b** Sensitivity estimation using semi-simulated data. We mixed reads from a cell line and reads from an unmatched normal sample to mimic decreasing tumor purity. Variants genotyped by SNP arrays in the pure cell line sample were used as answers. **c** Isolated calls, i.e., variants called by each given tool, and those not called by the given tool but by all the others, in 70 FFPE samples. Target regions were the same between all the tools and the numbers were normalized by the target region size. **d** SC-FPs and SC-FNs evaluated by mass spectrometry for variants from the datasets of panel **c**. The sample size (*n*) is indicated below the *x*-axis. Variants with ≥ 5% VAFs were selected. **e** Integrative Genomics Viewer (IGV) [33] screenshot of an SNV that was called by both cisCall and mass spectrometry but missed by Mutect

We evaluated the performance of cisMuton in comparison with Mutect [9], Shearwater [23], Varscan2 [10], and Strelka [11]. We first evaluated these tools using controlled negative/positive data. As negative data, the same tissue block was used to extract tumor FFPE samples for foreground data and tumor frozen samples for background data. Here, not FFPE but frozen samples were used as the background because we assumed unavailability of non-pathological FFPE samples in *actual* clinical sequencing. The extracted FFPE and frozen samples theoretically have the same tumor mutations; therefore, any calls from these data should be false positives in the FFPE samples. cisMuton and Mutect yielded no calls (Fig. 1a). Shearwater and Varscan2 reported some (2 and 10 per 477 kb target size) calls, whereas Strelka generated ~ 1000 calls per 477-kb target size (Fig. 1a).

For the false-negative rate, we used semi-simulated data as positive data because it is difficult to know all variants in natural samples. We randomly mixed reads from a lung cancer cell line (100% tumor purity) with reads from an unmatched normal sample to mimic a wide range of tumor purity. The depth was 970 on average, where we aimed for a depth of 1000 because our power calculation suggested that this would be ideal. We used these mixed datasets and a different normal dataset as the fore- and background datasets for calling, respectively. Variants genotyped by SNP arrays in the pure cell-line sample were used as answers. cisMuton showed 100% sensitivity for cell line/normal sample ratios down to 10%, and nearly 80% sensitivity at a ratio of 5% (Fig. 1b). Strelka showed the same sensitivity, but the other tools performed less well. The specificity was almost the same (~ 1) among all the tools. Based on both the negative control and these positive control results, cisMuton demonstrated the best performance.

Next, we made calls for 70 FFPE samples (Additional file 2: Figure S4). For each tool, we counted the number of variants called, as well as those not called by the given tool but detected by all other tools, because we considered that such isolated calls would reflect the specific nature of each algorithm. cisMuton reported the least isolated SNVs and indels, indicating that it was the most balanced among all tools (Fig. 1c). For reference, the numbers of variants removed by the noise filters are shown in Additional file 2: Table S4. cisMuton’s calls together with their VAFs and histologically determined tumor purity are shown in Additional file 2: Figure S5.

Variants with low VAFs (such as < 10%) are recommended to be filtered out in sequencing of FFPE samples [32]. Because our criterion in this clinical sequencing was to select FFPE samples with a pathologically measured tumor purity of ≥ 10%, we stratified the isolated calls at 5% VAFs (assuming the maximum major clone in the copy number neutral state). Even taking into account ≥ 5% VAFs, substantial numbers of isolated calls were found in all of the other tools for SNVs, and cisMuton was the most balanced (Fig. 1c). For indels, cisMuton was not the most balanced anymore; many (23 per 477 kb target size) indels were called only by cisMuton. Nevertheless, validation analysis, as described below, should be needed for such isolated calls, which may be false negatives for the other tools.

We selected variants from isolated calls in Fig. 1c for validation by mass spectrometry (Sequenom MassARRAY), where calls with ≥ 5% VAFs were selected because of difficulty in detecting variants with lower VAFs by mass spectrometry, and because of our criterion to select FFPE samples with a tumor purity of ≥ 10%. We refer to false positives/negatives in this isolated-call validation as severe conditioned-false positives/negatives (SC-FPs and SC-FNs) because isolated calls were expected to be less validated than the other calls such as those called by multiple tools. We compared the performance of cisMuton with that of Mutect and Strelka for SNVs and indels, respectively. Whereas cisMuton yielded 1/16 SC-FPs and 2/13 SC-FNs, Mutect generated 15/16 SC-FPs and 11/13 SC-FNs (Fig. 1d). An Integrative Genomics Viewer [33] screenshot of mass spectrometry-validated SNVs that were called by cisCall but missed by Mutect shows substantial noise *around* the SNV that was supposed to come from FFPE processes (Fig. 1e). For deletions, cisMuton yielded no SC-FNs, whereas Strelka generated 4/4 SC-FNs. Both tools did not greatly differ (one or zero counts) in SC-FP in deletions and SC-FP/FN in insertions.

We examined factors that may be associated with performance. 1) SC-FP and SC-FN variants seemed to be affected by depth and tumor purity. The effective depth, defined by the depth of each variant × pathologically measured tumor purity, was low for the SC-FP variants in cisCall (median 26.0, compared with 184.5 for the other variants) and in Mutect (29.7) (plotted in Additional file 2: Figure S6). The effective depth was low for the SC-FN variants in cisCall (53.1) and high for those of Mutect (503.1), probably due to excess filtering as shown above in Fig. 1e. 2) The FFPE storage time seemed to affect the performance. SC-FP tended to be found in older FFPE samples (median 53.0 and 55.0 months over samples with SC-FP variants for cisCall and Mutect, compared with 24.5 months over the other samples; Additional file 2: Figure S6). 3) Mutation load did not seem to be related to SC-FP and SC-FN (Additional file 2: Figure S6), although the range of the number of mutations was insufficient in this study (5–20 somatic mutations detected in 90% of the samples).

### Fusion calling

#### Features of cisFusion

cisFusion detects gene fusions and their breakpoint positions in either single-end or paired-end targeted DNA sequencing reads. In contrast, most existing tools have been developed for RNA-seq or paired-end whole-genome sequencing [18]. As RNA is more prone to degradation than DNA, we used DNA for fusion calling from the FPPE samples. Because most gene fusions occur in intron regions [25], we designed capture regions to include such introns. cisFusion utilizes local alignment (BWA-SW [34]) to easily extract breakpoint positions; in contrast, many other callers primarily use global alignment, in which additional complex procedures such as splitting reads are usually required for extracting breakpoint positions. Please refer to the “Methods” and Additional file 2: Text S1 for the algorithmic details. cisFusion typically takes 1 h 52 min ± 35 min (s.d.) using the same settings described above in the cisMuton section.

#### Performance evaluation of cisFusion

**Fig. 2:**
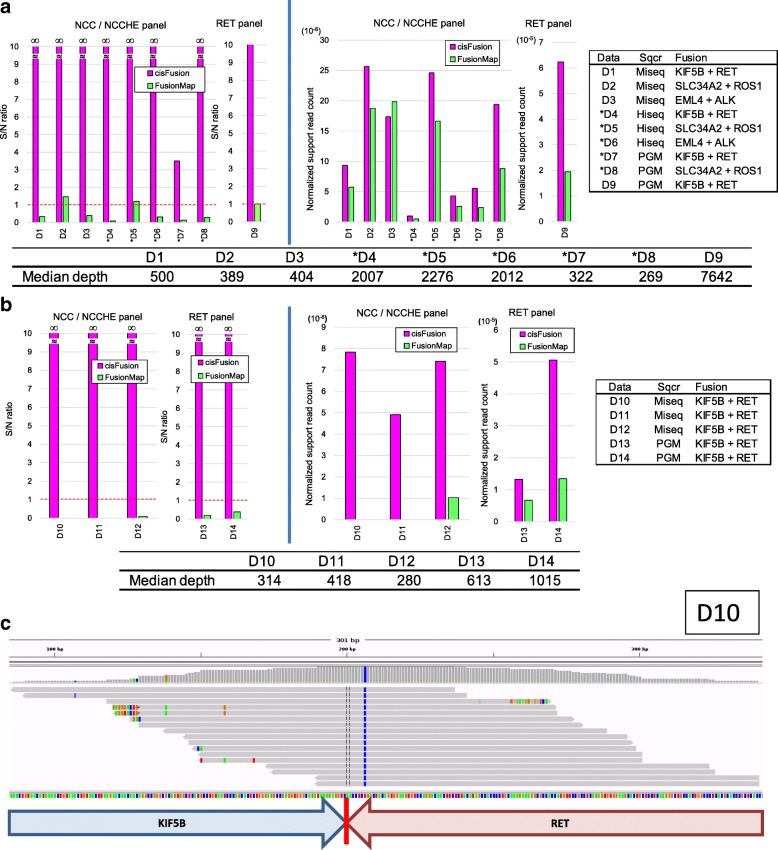
cisFusion calls. cisFusion evaluation for cell lines and frozen clinical samples (**a**) and for FFPE clinical samples (**b**). The *y*-axis represents the signal-to-noise (*S/N*) ratio: the ratio of the number of support reads for a correct fusion gene to the number of support reads for an incorrectly detected fusion candidate with the largest number of support reads. S/*N* > 1, shown with the *red broken line*, indicates that correct fusions are ranked at the top. The normalized support read count in the *y*-axis represents the number of support reads for a correct fusion divided by the number of all mapped reads. The *asterisks* indicate datasets where the target panels were designed to capture one gene of a fusion pair; otherwise, the panels were designed to capture both genes. The details of datasets and panels are presented in Additional file 1: Table S1. *Sqcr* sequencer. **c** IGV [33] screenshot showing an example of support reads in an FFPE sample for a fusion detected by cisFusion but missed by FusionMap

Because the prevalence of fusion genes in actual clinical samples is very low [35], we used cell lines (*n* = 5), frozen tissue (*n* = 4), and FFPE tissue samples (*n* = 5) known to contain fusion genes (Additional file 1: Table S1). We compared cisFusion with FusionMap [16], which is also applicable to DNA target sequencing using single- and paired-end reads. In all 14 datasets, cisFusion ranked correct fusions as the top candidate, as indicated by signal-to-noise (S/N) ratios of more than one (Fig. 2a, b). cisFusion yielded no false positives in all but one case, as indicated by S/N ratios noted as infinity. In contrast, FusionMap ranked incorrect fusion candidates as the top candidate in 12 of the 14 samples, as indicated by S/N ratios of less than one. In two of the five FFPE samples (Fig. 2b), FusionMap did not even list correct fusions, as indicated by S/N ratios of zero. Fig. 2c shows an example of support reads of a fusion gene in an FFPE sample that was detected by cisFusion but not by FusionMap. Mismatch bases are substantially observed. The normalized support read count (Fig. 2a, b) indicates that cisFusion demonstrated better sensitivity than FusionMap in all cases except one. In particular, remarkable superiority in both the specificity (the S/N ratio) and sensitivity was observed in FFPE samples. Additionally, fusion breakpoints were correctly predicted in 9/9 frozen and 3/5 FFPE cases by cisFusion, and in 8/9 frozen and 1/5 FFPE cases by FusionMap (although with some differences in base pairs; Additional file 4: Table S5).

### CNA calling

#### Features of cisCton

**Fig. 3:**
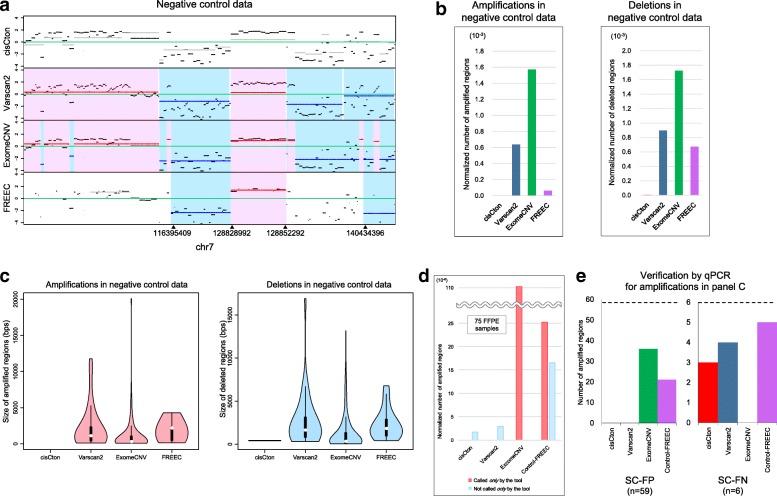
cisCton calls**. a** Log ratio values and segmentation in negative control data for an FFPE sample. *Red* and *blue regions* indicate regions called as amplifications and deletions, respectively, despite the data being negative control data. **b** Normalized numbers of regions called as amplified or deleted out of the target capture regions in the negative control data of two FFPE samples. The normalization was based on target region size (477 k bp) for possible comparison with other gene panels with different sizes. No calls are expected in these negative control data. **c** Violin plots to show the sizes of consecutive regions in panel **b**. **d** Isolated calls, i.e., regions called by each tool, and those not called by the given tool but called by all the other tools, for 75 FFPE samples. The normalization was based on the target region size (477 k bp). **e** Numbers of SC-FPs and SC-FNs confirmed by qPCR for 65 calls randomly selected from the datasets in panel **c**. The sample size (*n*) is indicated below the *x*-axis

cisCton discovers CNAs in targeted sequencing data on the basis of the log ratio of the read depth of a tumor sample to that of a control sample. cisCton utilizes a non-parametric statistic in the circular binary segmentation (CBS) framework [36] and a process to abort detected segments with high fluctuations to manage the strong noise in FFPE data, as shown in Fig. 3a. Details of the algorithm are described in the Methods and Additional file 2: Text S1. cisCton typically takes 1 h 55 min ± 6 min (s.d.) using the same settings described above in the cisMuton section.

#### Performance evaluation of cisCton

We first evaluated false positives using the same negative control data as used in SNV/indel calling. Performance was compared with that of Varscan2 [10], ExomeCNV [19], and Control-FREEC [20], which can call somatic CNAs from targeted (or exome) sequencing data by the read-depth method [21]. For a fair comparison, we counted the number of amplified or deleted target-capture regions. In the negative control data, cisCton called no amplified regions and almost no (3 per 477-kb target size) deleted regions (Fig. 3b, c). The other tools called hundreds of false amplified and deleted regions per 477-kb target size (Fig. 3b, c).

Next, we ran the tools on the 75 FFPE samples. The Venn diagrams of the calls are shown in Additional file 2: Figure S7. In clinical sequencing, molecularly targeted drugs are usually applied to amplifications; thus, we focused on amplifications. For each tool, we counted isolated calls, i.e., amplified regions called by the given tool, and those called by the other tools but not the given tool. cisCton had the lowest count of isolated calls, whereas ExomeCNV and Control-FREEC yielded approximately 100 isolated calls (Fig. 3d). From isolated calls made at the gene level, we randomly selected 65 calls for which qPCR probes were successfully designed. qPCR experiments revealed that cisCton was most balanced for SC-FPs (0/59) and SC-FNs (3/6) (Fig. 3e). ExomeCNV had many SC-FPs (36/59) but no SC-FNs (0/6). Control-FREEC yielded 21/59 SC-FPs and 5/6 SC-FNs. Varscan2 yielded the same number of SC-FPs (0/59) as cisCton but slightly more SC-FNs (4/6).

## Discussion

We developed an SNV/indel/fusion/CNA calling tool specialized for data obtained from FFPE samples. cisCall was previously employed in clinical sequencing for a clinical study [24], in which a good validation rate of 128/129 for SNVs and 12/13 for indels in 70 samples was confirmed by mass spectrometry. We also used a commercially available FFPE reference material and successfully detected all eight SNVs with > 5% VAFs, further obtaining a good concordance between expected and computed VAFs (*R* = 0.99; Additional file 2: Figure S8), although the specificity cannot be evaluated in this type of material because not all variants are known in advance. We conducted a rigorous tool comparison using SC-FPs and SC-FNs based on isolated calls; however, it is worth noting that these numbers would be inflated compared with the validation rate based on all calls. A more rigorous way to evaluate the performance would be to 1) prioritize samples, 2) call alterations by tools, 3) validate all alterations called by any of the tools based on orthogonal methods such as mass spectrometry and qPCR, and 4) calculate evaluation indices such as specificity, sensitivity, and F-measure. We demonstrated that cisCall outperformed currently available tools developed for exploratory research purposes, which generally assume the use of cell lines or fresh-frozen clinical samples. One caveat is that we used default parameters for the compared tools; we did not use *LOD*_T_ of > 50 for Mutect, which is recommended for FFPE samples [37].

We reason that our tool performed well because 1) we used non-parametric statistical methods wherever possible to absorb abrupt, unpredictable fluctuations stemming from FFPE errors, and 2) we elaborated noise filters for all types of mutations, such as the misalignment filter for SNVs/indels and the abortion filter for CNAs. We believe that this design concept also makes cisCall applicable to reads from Ion sequencers. When we evaluated the performance of cisMuton for Ion PGM data using semi-simulated data, it showed the highest (100%) sensitivity among all tested tools for cell line/normal mixtures down to 10% cell line ratios (Additional file 2: Figure S9; the specificity was ~ 1 for all tools).

Commercially developed algorithms for detecting SNVs/indels and CNAs from FFPE-based sequencing data have been reported [6]; however, the software is not publicly available. Moreover, regarding SNVs, the software performance was systematically evaluated mainly using cell lines, the sample features of which are more similar to frozen than to FPPE clinical samples, and based on germline, not tumor, variants [6]. In addition, the fusion algorithm was not systematically evaluated. Another research group reported an in-house SNV/indel-calling program fine-tuned for FFPE samples [38]; however, the detailed algorithm was not described, and the tools were not subjected to systematic comparison with other tools. In contrast, we evaluated all SNV/indel/CNA/fusion calling algorithms in FFPE samples. Our software tool is publicly available.

Although cisCall showed the best performance on SC-FN in CNA evaluation (Fig. 3), the SC-FN rate was relatively high. The reason for this was the discrepancy in the strength of GC-content correction between FFPE and frozen samples. Depth values fluctuated more in foreground FFPE samples than in the background frozen sample. Depth values in regions with high GC content in the FFPE samples scattered up to high values. Because there were only a few high GC content regions in our targeted regions, the LOWESS curve was easily pulled upward. GC-content correction was thus weaker in FFPE samples than in frozen samples for regions with high GC content, and hence amplification signals in FFPE samples were cancelled out. cisCall failed to call CNAs in high GC-content regions. It is necessary to improve the LOWESS procedure for regions with high GC content. Also, use of control FFPE samples will be another possibility to improve the baselines for CNA detection.

The limitations of our algorithms are: 1) large indels are not targeted, i.e., we assumed indels with BWA-mapped sizes (≤ 6 bps found in our cases); 2) we did not assume whole-exome or whole-genome sequencing; and 3) consequently, we cannot handle structural variations beyond targeted fusion genes. We are currently working on overcoming these limitations. For application to different experimental settings, such as whole-exome sequencing, elaborate parameter tuning will be necessary. Additionally, application to other gene panels should be tested and the algorithms and codes should be improved for faster computation.

Because matched normal samples were not available in this project, we evaluated our tool in tumor–mixed normal paired samples. To filter out germline SNPs, we removed mutations listed in SNP databases and those with 40–60% and > 96% VAFs. This simple approach can largely remove germline SNPs and a more sophisticated approach using machine learning may be possible [39]; however, it is desirable to use matched normal samples for precise filtering if such samples are available. It is in principle possible to apply our tool to tumor–matched normal paired samples and we are obtaining preliminary results in such samples, though further investigation should be needed. Off-target reads that mapped on non-target regions constituted more than 0.1× coverage (0.3–0.4× on average) genome-wide in our data; utilization of these reads may help identifying copy-number loss related to homologous recombination repair deficiency in non-target regions in FFPE samples [40, 41]. cisCall was developed for research purposes in clinical studies and is not intended for use in clinical tests regulated by the authorities, where analytical validity at the manufacturing level should be demonstrated. Nevertheless, these alteration-calling algorithms enable first steps in the translation of cancer clinical sequencing to everyday diagnostics.

## Conclusions

Clinical sequencing requires an accurate computational tool to call multiple types of DNA alterations—SNVs/indels, fusion genes, and CNAs—from NGS data in FFPE samples. We developed such a tool and demonstrated that our tool outperformed seven other tools that have been developed for explanatory research purposes. This is because our tool uses robust non-parametric statistics to select alteration candidates and more than ten elaborated noise filters that maximally utilize internal control values automatically calculated from observed data as inputs for the tool’s parameters so that the tool can efficiently remove inherent noise arising in FFPE samples that cannot be filtered out using other tools. Our tool allows us to accurately detect DNA alterations in multiple genes, which will promote more accurate and efficient cancer precision medicine.

## Additional files

Additional file 1: **Table S1.** Samples and datasets used in this study. (XLSX 12 kb)

Additional file 2: **Text S1, Tables S2 and S4, and Figures S1–S9.** (PDF 1210 kb)

Additional file 3: **Table S3.** TaqMan primer IDs used for validation of CNAs. (XLSX 10 kb)

Additional file 4: **Table S5.** Breakpoint positions predicted by cisFusion and FusionMap. (XLSX 11 kb)

## Abbreviations

CBS: Circular binary segmentation
CNA: Copy number alteration
CNV: Copy number variation
FFPE: Formalin-fixed paraffin-embedded
ICGC: International Cancer Genome Consortium
IGV: Integrative Genomics Viewer
LOWESS: Locally weighted scatterplot smoothing
NCC: National Cancer Center
NGS: Next-generation sequencing
PON: Panels of normal
S/N: Signal-to-noise
SC-FN: Severe conditioned-false negative
SC-FP: Severe conditioned-false positive
SNV: Single nucleotide variation
TCGA: The Cancer Genome Atlas
VAF: Variant allele frequency
