# Supplementary material for: A computational tool to detect DNA alterations tailored to formalin-fixed paraffin-embedded samples in cancer clinical sequencing
Source: Genome Med. 2018 Jun 7;10:44. doi: 10.1186/s13073-018-0547-0 (PMC5992758; doi:10.1186/s13073-018-0547-0)
Supplement: Supplementary file 2 — Text S1, Tables S2 and S4, and Figures S1–S9. (PDF 1210 kb) [file 13073_2018_547_MOESM2_ESM.pdf]

Text S1. Supplementary Methods

cisCall

cisCall accepts the FASTA format for the whole-genome reference sequence, the BED format for target capture regions, and FASTQ or BAM formats for sequence read data. cisCall typically takes in FASTQ/BAM for Illumina sequencers and BAM for Ion sequencers. When cisCall takes in FASTQ files, it uses built-in BWA [1] to internally maps reads for SNV/indels/CNAs, and uses BWA-SW [2] for fusions. Re-calibrated or re-aligned reads [3] can be loaded through BAM files.

cisMuton algorithm

The cisMuton algorithm consists of a series of prep filters, the variant extraction step, and noise filters as described below (outlined in Additional file 2: Figure S1).

- Prep filters: mapping-quality and base-quality filters

cisMuton uses read data to count the numbers of A, C, G, T, D (deletion), and I (insertion) at every position for a pair of tumor and normal samples. D and I are treated as entities with lengths (cisMuton does not count the same deletions and insertions multiple times). A normal sample may be unmatched. In counting, cisMuton filters out reads with low mapping qualities (default: MQ=0). Additionally, it drops bases with low base qualities (default: BQ<15). Insertions are filtered out if any of the inserted bases has a low base quality. Deletions are left out if either of the bases next to both ends of the deletion has a low base quality.

- Variant extraction step: Fisher's exact test

Using tumor-normal samples, cisMuton performs Fisher's exact test at each position for each of A, C, G, T, D, and I to screen for positions with a low *p*-value (default cut-off: a raw

p-value of 10^{-3}), a high proportion ratio (defined as the ratio of VAFs of a tumor to those of a normal sample; similar to an odds ratio) (default: 10), and more than a minimum number of counts in tumor and normal samples (default: 5 and 20, respectively). FPscore, which is –$\log_{10}$ of a p-value, as well as the proportion rate and variant counts are output in the output file of cisMuton.

Then, cisMuton proceeds to the first set of noise filters for extracted variants. Each variant is checked by each filter.

- Noise filter set 1: misalignment filter

This filter screens out calls for which the positions are correlated with other positions with read variants at close distances, where read variants are read bases different from a reference sequence used for alignment (not necessarily at called positions). According to our observations, a characteristic feature of misalignment is that read variants at one position are correlated with read variants at another position. They are often observed as multiple-color bands vertically running in parallel on Integrative Genomics Viewer (IGV) [4]. However, such a correlation may occur by chance; hence, cisMuton checks whether the number of occurrences of misalignment is statistically high.

Specifically, the algorithm first defines a correlation between two positions by multiple statistics based on Fisher's exact test using the number of co-occurrences of read variants at two positions. It then calculates a p-value by using a negative binomial distribution with a certain background probability for the number of occurrences of such correlations within a moving range of a called position (*e.g.*, up to ± 100 bps) and selecting the lowest p-value. If occurrences of correlations are statistically abundant, it discards the call.

- Noise filter set 1: strand-bias filter

This filter screens out calls with strand bias, i.e., where the number of reads mapped to the forward strand drastically differs from that of reads mapped to the backward strand. It uses

the proportion of mapped forward reads to all mapped reads as a strand bias index. We observed that values of the strand bias index were more broadly distributed than a binomial distribution at the background level; hence, we used a beta distribution to represent the background level. The algorithm estimates the shape parameters (α, β) of a beta distribution using the method of moments from the number of forward and backward reads at positions randomly sampled from the genome. This is regarded the background level of strand bias. Then, it performs a beta-binomial test for the number of forward and backward reads at a position of interest using the estimated beta distribution to filter out calls with low p-values.

- Noise filter set 1: within-long-homopolymer filter

This filter removes SNV/indel calls within long homopolymers of a reference sequence. Error (discordance) rates within homopolymers tend to drastically increase beyond a certain homopolymer length. To exclude calls within such long homopolymers, the algorithm first calculates discordance rates classified by homopolymers with different lengths for SNVs, insertions, and deletions. Then, it decides the length from which discordance rates drastically increase by selecting three consecutive lengths for which the discordance rates are a certain fold higher than the average discordance rate (if it does not find such a length, it uses a default, conservative length). Finally, it excludes calls within such long homopolymers.

- Noise filter set 1: MQ0 filter

This filter screens out calls for which the positions have too many reads with a mapping quality score of zero. We observed that erroneous calls had too many reads with a mapping quality of zero at their positions before the mapping quality filter was applied. For reads before exclusion by the mapping quality filter, it performs a binomial test using a certain success probability to remove such erroneous calls.

- Noise filter set 1: read-end-call filter

This filter removes SNV calls that tend to be located at read ends. It identifies reads with a variant called at the variant extraction step and then calculates the total number of read bases to the left and right of the called variant position. If the ratio of the number on the left to that on the right exceeds a certain threshold, the call is removed.

- Noise filter set 1: surrounded-by-dust filter

We observed in IGV that erroneous indel calls are often surrounded by mapped reads with many indels. Hence, if an indel rate within a certain range (*e.g.*, ± 100 bps) of a call position is statistically higher than the average overall reads in a binomial model, the algorithm discards the indel call.

- Noise filter set 1: abnormal-BQ-drop filter (for Ion-derived indels only)

For indels identified in Ion sequencing data, we observed that erroneous indel calls often had a number of indels that were dropped by our base-quality filter. Moreover, the dropping rate was more broadly distributed than expected from a binominal model; therefore, we use a beta distribution to model dropping rates. The algorithm estimates the shape parameters (α, β) of a beta distribution from the number of drops at positions randomly sampled from all positions. Using this estimated distribution as the background, it performs a beta-binomial test to detect and discard indel call positions with too many drops.

- Noise filter set 2: second Fisher filter

cisMuton further checks calls that pass all aforementioned filters. This filter screens out calls attributable to erroneous reads. It selects the reads that have no or few mismatches in the middle region as well as in their ends in foreground (tumor) data. To select for high-quality middle reads, it calculates the mismatch rate (with some modification: the numerator = the number of mismatches – 2; the denominator = a read length –6) of a read, excluding its 3-bp ends, and removes reads with high mismatch rates. To select for high-quality end reads, it calculates the mismatch rate within an extending window starting from a read end to any

position moving up to 1/4 of the read length. If it finds a position resulting in a mismatch rate of 2/3 or more, it trims bases from the read-end position to that position. If it finds multiple such positions, it uses the furthest position from the start. Using these clean reads, it performs the Fisher test again.

- Noise filter set 3: VAF-lees filter

cisMuton further filters out "lees" of calls that show suspiciously low VAFs but are not filtered for unknown reasons. Lees typically form a peak at a low value in the histogram of VAFs. cisMuton automatically detects a peak occurring at a low value and filters out calls with VAFs that are not significantly higher than the given peak.

Specifically, cisMuton represents a distribution of VAFs as a beta-mixture model and estimates values of model parameters using the expectation maximization algorithm [5]. It then calculates the ICL-BIC criterion [6] to select the best model of all models with different numbers (ranging from 1 to 10) of beta components. cisMuton then searches for the beta component for which the distribution's average is within a range of low frequencies (*e.g.*, 1–3%), which means that a peak of the VAFs is found at such a low value. If multiple peaks are found within the frequency range, cisMuton selects the component with the peak of the largest VAF. It regards such a component as an error distribution, and performs a beta-binomial test for variant and depth counts in a tumor sample to screen for calls with low *p*-values. Even if no peaks are found, cisMuton filters out calls with an extremely low VAF, using a binomial test with a success probability of a default value (*e.g.*, 1%).

cisFusion algorithm

The cisFusion algorithm entails 2map, VF, and paired-end steps. At the 2map step, cisFusion generates a local alignment between a query read sequence and every sequence from a reference set of genes (with introns), using BWA-SW [2]. Then, cisFusion filters out

reads with low mapping qualities and screens for reads mapped onto two different genes. cisFusion determines whether the entire span of reads is *exclusively* mapped on either gene. This is achieved by calculating the proportion of total read bases that are mapped only on either gene, and removing reads with low proportion values. Further, cisFusion evaluates whether the *left and right* ends of reads are *respectively* mapped onto two different genes, utilizing a p-value and the effect size of the Wilcoxon test for positions of bases mapped only on one gene and only on the other gene. Additionally, when control read data, such as data from a normal sample, are available, it selects gene pairs that specifically appear in a tumor sample, using a p-value and the odds ratio of Fisher's exact test for the number of reads mapped to a gene pair. When a read has multiple fusion candidates, cisFusion selects the best candidate with the highest mapping qualities. cisFusion extracts and counts possible breakpoints from local alignment results.

At the VF step, cisFusion first groups nearby breakpoints by taking up a breakpoint with the largest count and collecting breakpoints that are close (*e.g.*, ± 2 bps) on the coordinate of the one gene as well as on the coordinate of the other gene. cisFusion repeats this procedure for a breakpoint with the next largest count among remaining breakpoints until it visits all breakpoints to obtain multiple groups. In each group, cisFusion uses a breakpoint with the largest count to join two gene sequences together. Thus, cisFusion generates a virtual fusion.

cisFusion maps reads onto virtual fusions using BWA [1] and BWA-SW [2] to save reads that are initially unmapped because the alignment for one gene of a pair is too short. cisFusion filters out reads with low mapping qualities as well as reads with high base-mismatch rates in the alignment for either gene. cisFusion then checks if reads are exclusively mapped to either gene; it filters out reads if read bases have few matches (*e.g.*, <5) with bases unique to either gene. Finally, it reports the aggregated number of support reads at the 2map and VF steps as well as breakpoint positions for each gene pair.

In addition to single-end reads, cisFusion can handle paired-end reads (Additional file 2: Figure S2). Calls at the 2map and VF steps only rely on the presence of a breakpoint *within* single-end reads, whereas calls at this step rely on a breakpoint *between* paired-end reads. In the paired-end mode, cisFusion first separates one ("R1") and the other ("R2") of paired reads to run the same procedure as the single-end mode. Then, cisFusion filters out reads with low mapping qualities and reads with high mismatch rates, and searches for evidence on a breakpoint between R1 and R2 by detecting R1 and R2 mapped to different genes in a local alignment. We confirmed that local alignment was better than global alignment at this step. When a paired-end read has multiple fusion candidates, it selects the best candidate based on the highest mapping qualities.

If cisFusion detects possible breakpoints *within* R1 or R2, it uses the same procedure as the 2map and VF steps to extract breakpoint positions. Otherwise, cisFusion utilizes detected paired-end reads to reinforce evidence on breakpoint positions identified by VF calling. That is, cisFusion calculates the distances from the borders of mapped paired-end read bases to (multiple) breakpoint positions suggested by VF calling and selects the breakpoint for which the total distance over both genes is the closest to the average insert size of paired reads and within a reasonable distance (the median insert size plus 3 standard deviations). Thus, such a VF-identified breakpoint is regarded to be supported by paired-end reads, and cisFusion augments the number of reads that supports the breakpoint. cisFusion finally reports the number of support reads and breakpoint positions for each gene pair. In the paired-end mode, R1 and R2 are counted as one support read, whereas reads are counted separately in the single-end mode.

cisFusion makes the final call based on thresholds for the number of support reads normalized to that of mapped reads, the balance between 2map and VF support reads, etc.

cisFusion outputs files that can serve as input for IGV [4] to visually confirm virtual fusions and mapped reads.

cisCton algorithm

cisCton comprises the following six steps: 1) bin definition, 2) GC-content correction, 3) segmentation, 4) stitching of split windows for chromosome-size segmentation, 5) abortion of abnormal segments, and 6) amplification/deletion decision. At the first step, cisCton defines bins by dividing target capture regions by the median size of all capture regions such that long capture regions for introns become short enough for easy handling. It uses the median depths within bins as the minimum unit value for CNA segmentation (to avoid biases caused by lower depths at the ends of capture regions).

We observed that depths were biased by GC contents up to 10 kb around capture regions. To correct for this bias, cisCton performs locally weighted scatterplot smoothing (LOWESS) regression between the bin depths and GC contents flanking 10 kb from the middles of the bins for a tumor and a normal sample and then divides depths by LOWESS predicted values as follows:

$$d_{\mathrm{GC}} = (d_{\mathrm{raw}} / d_{\mathrm{LOWESS}}) \times d_{\mathrm{target}} \quad (1)$$

where d_{GC}, d_{raw}, d_{LOWESS}, and d_{target} represent GC-corrected, raw, LOWESS-predicted, and target-region average (median) depths.

At the segmentation step, cisCton uses circular binary segmentation (CBS) with correction of the edge effect [7] for log$R$:

$$\log R = \frac{d_i^{\mathrm{T}} / d_{\mathrm{base}}^{\mathrm{T}}}{d_i^{\mathrm{N}} / d_{\mathrm{base}}^{\mathrm{N}}} \quad (2)$$

where d_i is the depth of bin i and d_{base} is the median depth over baseline regions, and T and N represent tumor and normal samples. cisCton detects deviations from baseline regions, which

are defined by target regions with a mappability of one in the default setting under the assumption that most targeted regions are copy-number neutral. Alternatively, users can specify their own baseline regions such as regions (*e.g.*, no known germline CNVs or somatic CNAs) that we specially designed in the latest version of the gene panel. The difference in CBS from the previous study [7] is that cisCton uses a non-parametric statistic (the Mann–Whitney U statistic) for dealing with noisy fluctuations in FFPE samples rather than the essentially Gaussian statistic used in the previous study. A naive CBS requires a long computation time for a chromosome-level size; therefore, cisCton splits a chromosome into windows of a specified size that slide along the chromosome by half of the window size, such that the end of each window overlaps with the middle of the neighboring window (because segmentation tends to be inaccurate at the window ends). cisCton performs CBS within each window. At the stitching step, it stitches neighboring segments in overlapping windows using the same algorithm as for the edge effect correction [7].

We observed in our FFPE samples that the variance of log$R$ values around the log$R$ median across a segment was occasionally too high, indicating inadequate segmentation. Hence, we introduced the abortion step, where if the proportion of the number of log$R$ values deviating from the log$R$ median in a segment exceeds a certain threshold, cisCton aborts the segment and treats all of the log$R$ values in a segment as independent point values.

Finally, cisCton defines amplifications or deletions by a bootstrapping approach in which it randomly selects the same number of log$R$ values from baseline regions as that of an actual segment, and compares the observed log$R$ median with log$R$ medians calculated from the bootstrapped log$R$ values to obtain a p-value.

SNV/indel evaluation

For the FFPE samples

We ran cisMuton (ver. 5), Mutect [8], Shearwater [9], Varscan2 [10], and Strelka [11] using default parameters. We selected these tools because they were developed to identify somatic mutations in tumors, taking in a foreground dataset from a tumor sample and a background dataset from a normal sample to well differentiate tumor variants from germline variants or noise. For Mutect, we did not use a panel of normal samples (PON) filter as we were unable to collect individual normal (non-pathological) samples. For Strelka, BWA configurations were used and the depth filter was turned off per the authors' recommendation. FASTQ data were mapped to hg19 gene sequences (including intron regions) in the target BED file for fast calculation. Mapped BAM files were used as input for each of the variant callers. Only calls located in target capture regions were considered. We excluded sites of known germline SNPs (1000 Genomes, http://www.1000genomes.org; ESP6500, http://evs.gs.washington.edu/EVS; Human Genetic Variation Database, http://www.genome.med.kyoto-u.ac.jp/SnpDB; and in-house Japanese germline SNP data) and those with VAFs of 40–60% and 96–100% because of the possibility of personal SNPs. In validation, we further excluded homo-polymer (≥ 6-mer) and microsatellite regions because of difficulties in probe design for mass spectrometry.

For semi-simulated data

We randomly mixed read data of a lung-cancer cell line (HCC78) with read data of an unmatched noncancerous sample to obtain a same total number of reads as the original cell line data, with a desired ratio of cell line to normal sample data. The noncancerous DNA was obtained from normal lung tissues of lung cancer patients who underwent lobectomy. We sequenced these samples by HiSeq (Illumina, CA, USA) as well as Ion PGM (Thermo Fisher Scientific, MA, USA). We used the NCC oncopanel v1 as the target gene panel. We ran the

five variant callers as described above (cisMuton has a parameter set for frozen and cell line samples; we used it). Note that we used an unmatched sample as the background set because we wished to obtain sufficient variants; consequently, these may be mostly germline variants.

We compared calls from these NGS data with microarray genotyping calls used as answers to calculate the sensitivity. We genotyped both the lung cancer cell line and the normal samples using the Infinium Omni2.5-8 microarray (Illumina, CA, USA) per the manufacturer's instructions. The plots of log*R* ratio and BAF (B allele frequency) were visually inspected to exclude CNA regions. We used only 330 loci (overlapping with target capture regions) at which genotypes of the normal sample were homozygous to avoid unnecessary complications.

Fusion evaluation

Because fusion genes are rare, we prepared samples previously revealed to have fusion genes [12-15], which we confirmed by PCR. No more than one fusion gene was identified in each sample. We ran FusionMap [16] using hg19 as the Genome Reference Library and RefGene as the Gene Model. For the S/N ratio and normalized support read count, we counted seed/rescued counts and 2map/VF counts as support read counts in FusionMap and cisFusion (ver. 5), respectively.

CNA evaluation

We ran Varscan2 [10], ExomeCNV [17], and Control_FREEC [18] for comparison with cisCton (ver. 5). These tools use depth information to identify tumor CNAs using a foreground dataset from a tumor sample and a background dataset from a normal sample. Specifically, we used BWA [1] to map reads to the human genome. BWA randomly maps reads only once when multiple mapping is possible; hence, we used all mapped reads for

CNA calling. From BWA-mapped BAM files, we ran ExomeCNV according to the pipeline recommended by the authors. Similarly, we ran Varscan2 and Control-FREEC per the authors' recommendation. Some tools generate segments seamless along chromosomes, and others generate discrete long segments as output. For a fair comparison, we intersected segments called by each tool with target capture regions in the BED file of the NCC oncopanel and determined the numbers and sizes of those intersected regions, excluding long regions for fusion capture.

Next, we counted capture regions called as amplifications in one tool but not in the other tools, or vice versa, for FFPE samples. Here, we only considered amplifications with strong intensities defined as $\log_2$ ratio values > 2 in the amplified segments. Then, we randomly selected several genes that overlapped with the amplified regions for TaqMan qPCR experiments. We selected genes, not the amplified regions themselves, because the TaqMan probes were defined for genes. We examined qPCR probe regions to determine whether amplification calls (with NGS $\log_2$ ratio values > 2) from a CNA tool coincided with amplification calls (with qPCR $\log_2$ ratio values > 2) in qPCR with a small difference in the $\log_2$ ratio values between NGS and qPCR (< 1 in the plus direction if qPCR indicated no amplification; <1 in the minus direction if qPCR indicated amplification).

References

1. Li H, Durbin R: **Fast and accurate short read alignment with Burrows-Wheeler transform.** *Bioinformatics* 2009, **25:**1754-1760.
2. Li H, Durbin R: **Fast and accurate long-read alignment with Burrows-Wheeler transform.** *Bioinformatics* 2010, **26:**589-595.
3. DePristo MA, Banks E, Poplin R, Garimella KV, Maguire JR, Hartl C, Philippakis AA, del Angel G, Rivas MA, Hanna M, et al: **A framework for variation discovery and genotyping using next-generation DNA sequencing data.** *Nat Genet* 2011, **43:**491-498.
4. Robinson JT, Thorvaldsdottir H, Winckler W, Guttman M, Lander ES, Getz G, Mesirov JP: **Integrative genomics viewer.** *Nat Biotechnol* 2011, **29:**24-26.
5. Ji Y, Wu C, Liu P, Wang J, Coombes KR: **Applications of beta-mixture models in bioinformatics.** *Bioinformatics* 2005, **21:**2118-2122.
6. Biernacki C, Celeux G, Govaert G: **Assessing a mixture model for clustering with the integrated completed likelihood.** *Ieee Transactions on Pattern Analysis and Machine Intelligence* 2000, **22:**719-725.
7. Olshen AB, Venkatraman ES, Lucito R, Wigler M: **Circular binary segmentation for the analysis of array-based DNA copy number data.** *Biostatistics* 2004, **5:**557-572.
8. Cibulskis K, Lawrence MS, Carter SL, Sivachenko A, Jaffe D, Sougnez C, Gabriel S, Meyerson M, Lander ES, Getz G: **Sensitive detection of somatic point mutations in impure and heterogeneous cancer samples.** *Nat Biotechnol* 2013, **31:**213-219.
9. Gerstung M, Papaemmanuil E, Campbell PJ: **Subclonal variant calling with multiple samples and prior knowledge.** *Bioinformatics* 2014, **30:**1198-1204.
10. Koboldt DC, Zhang Q, Larson DE, Shen D, McLellan MD, Lin L, Miller CA, Mardis

ER, Ding L, Wilson RK: **VarScan 2: somatic mutation and copy number alteration discovery in cancer by exome sequencing.** *Genome Res* 2012, **22:**568-576.

11. Saunders CT, Wong WS, Swamy S, Becq J, Murray LJ, Cheetham RK: **Strelka: accurate somatic small-variant calling from sequenced tumor-normal sample pairs.** *Bioinformatics* 2012, **28:**1811-1817.

12. Mizukami T, Shiraishi K, Shimada Y, Ogiwara H, Tsuta K, Ichikawa H, Sakamoto H, Kato M, Shibata T, Nakano T, Kohno T: **Molecular mechanisms underlying oncogenic RET fusion in lung adenocarcinoma.** *J Thorac Oncol* 2014, **9:**622-630.

13. Nakaoku T, Tsuta K, Ichikawa H, Shiraishi K, Sakamoto H, Enari M, Furuta K, Shimada Y, Ogiwara H, Watanabe S, et al: **Druggable oncogene fusions in invasive mucinous lung adenocarcinoma.** *Clin Cancer Res* 2014, **20:**3087-3093.

14. Kohno T, Ichikawa H, Totoki Y, Yasuda K, Hiramoto M, Nammo T, Sakamoto H, Tsuta K, Furuta K, Shimada Y, et al: **KIF5B-RET fusions in lung adenocarcinoma.** *Nat Med* 2012, **18:**375-377.

15. Seki Y, Mizukami T, Kohno T: **Molecular Process Producing Oncogene Fusion in Lung Cancer Cells by Illegitimate Repair of DNA Double-Strand Breaks.** *Biomolecules* 2015, **5:**2464-2476.

16. Ge H, Liu K, Juan T, Fang F, Newman M, Hoeck W: **FusionMap: detecting fusion genes from next-generation sequencing data at base-pair resolution.** *Bioinformatics* 2011, **27:**1922-1928.

17. Sathirapongsasuti JF, Lee H, Horst BA, Brunner G, Cochran AJ, Binder S, Quackenbush J, Nelson SF: **Exome sequencing-based copy-number variation and loss of heterozygosity detection: ExomeCNV.** *Bioinformatics* 2011, **27:**2648-2654.

18. Boeva V, Zinovyev A, Bleakley K, Vert JP, Janoueix-Lerosey I, Delattre O, Barillot E:

Control-free calling of copy number alterations in deep-sequencing data using GC-content normalization. *Bioinformatics* 2011, **27:**268-269.

Table S2. Genes in the NCC oncopanel v2.

a

Exon-targeted genes					
ABL1	*BRCA2*	*EZH2*	*JAK3*	*NOTCH1*	*RAC2*
AKT1	*CCND1*	*FBXW7*	*KEAP1*	*NOTCH2*	*RAD51C*
AKT2	*CDK4*	*FGFR1*	*KIT*	*NOTCH3*	*RAF1*
AKT3	*CDKN2A*	*FGFR2*	*KRAS*	*NRAS*	*RB1*
ALK	*CHEK2*	*FGFR3*	*MAP2K1*	*NRG1*	*RET*
APC	*CREBBP*	*FGFR4*	*MAP2K4*	*NT5C2*	*ROS1*
ARID1A	*CTNNB1*	*FLT3*	*MAP3K1*	*PALB2*	*SETD2*
ARID2	*CUL3*	*HRAS*	*MAP3K4*	*PBRM1*	*SMAD4*
ATM	*DDR2*	*IDH1*	*MDM2*	*PDGFRA*	*SMARCA4*
AXIN1	*EGFR*	*IDH2*	*MET*	*PDGFRB*	*SMO*
BAP1	*ENO1*	*IGF1R*	*MTOR*	*PIK3CA*	*STAT3*
BARD1	*EP300*	*IGF2*	*MYC*	*PIK3R1*	*STK11*
BCL2L11	*ERBB2*	*IL7R*	*MYCN*	*PTCH1*	*TP53*
BRAF	*ERBB3*	*JAK1*	*NF1*	*PTEN*	*TSC1*
BRCA1	*ERBB4*	*JAK2*	*NFE2L2*	*RAC1*	*VHL*

b

Kinases	Partners	
AKT3	*AGTRAP*	*KIF5B*
ALK	*C2orf44*	*KLC1*
BRAF	*CCDC6*	*MAGI3*
EGFR	*CD74*	*SDC4*

ERBB4	*CIT*	*SEC16A*
FGFR2	*EML4*	*SLC34A2*
FGFR3	*EPB41*	*SLC45A3*
NOTCH1	*ESRP1*	*TACC3*
NRG1	*EZR*	*TPM3*
RAF1	*FN1*	*TPM4*
RET	*GOPC*	*VCL*
ROS1	*KIAA1549*	

This panel included 90 exon-targeted genes and 35 fusion genes (12 kinases and 23 partners). The total size of the target capture regions was 931 kb. **a** Exon-targeted genes. The total size of the capture regions was 477 kb. **b** Fusion genes. The total size of the capture regions was 454 kb.

Table S4. Numbers of variants removed by the noise filters.

Set number	Noise filter	Number of variants
Set 1	Strand-bias	5,974
	Read-end-call	2,220
	Within-long homopolymer	501
	MQ0	19
	Surrounded-by-dusts	3,437
	Misalignment	2,109
Set 2	Second Fisher	5,292
Set 3	VAF-lees	4,486

In filter set 1, a variant was removed when flagged by at least one filter.

Figure S1.

a

- Prep filters: mapping-quality and base-quality filters
- Variant extraction: Fisher's exact test
  - *P*-value, proportion ratio (~odds ratio), FG count
- Noise filter set 1
  - Misalignment filter
  - Strand-bias filter
  - Within-long homopolymer filter
  - MQ0 filter
  - Read-end-call filter
  - Surrounded-by-dust filter
  - Abnormal-BQ-drop filter (for Ion indels only)
- Noise filter set 2
  - Second Fisher filter
    - Erroneous-read filter and trimming filter
- Noise filter set 3
  - VAF-lees filter

b

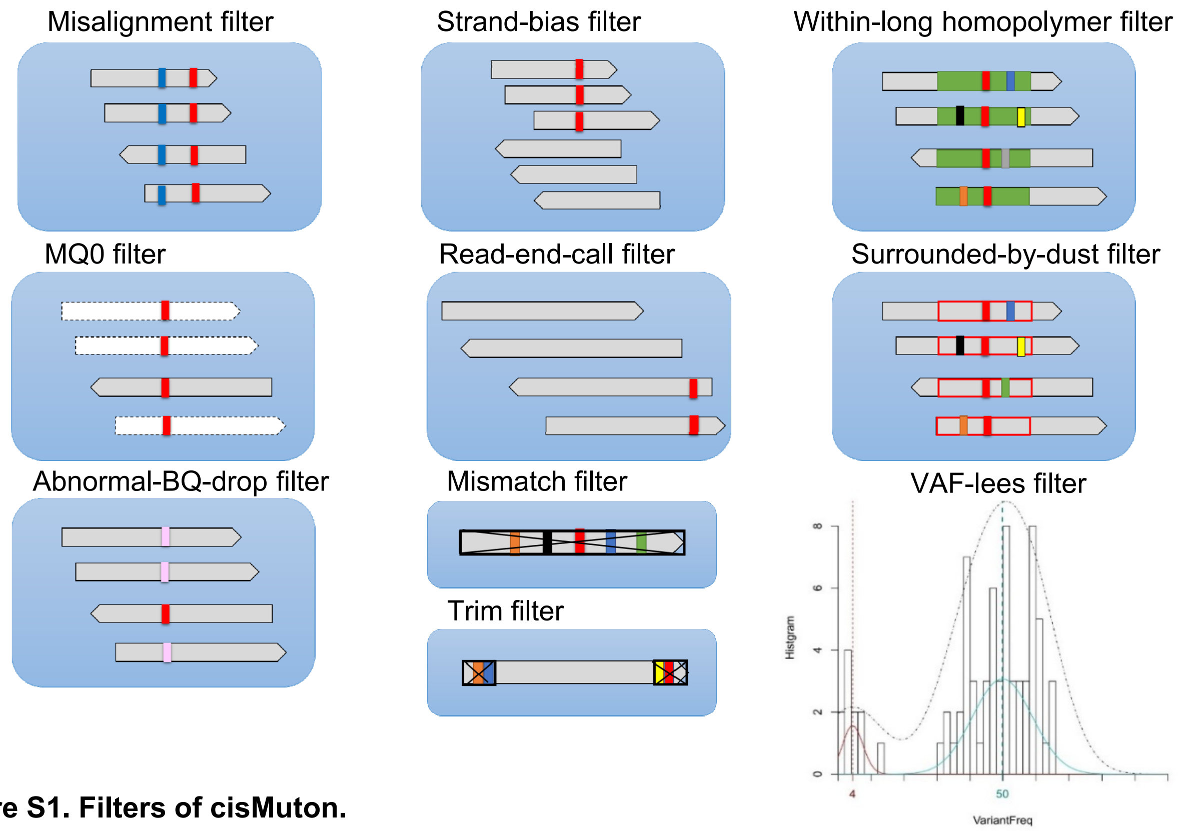

Figure S1. Filters of cisMuton.
a Filters. Refer to Additional file 2: Text S1 for details. **b** Illustration of filters. Vertical lines in reads represent mismatches. The green bands in reads for the within-long homopolymer filter represent long homopolymers. The red frames in reads for surrounded-by-dust filters represent regions in which "dust" was counted. For mismatch and trim filters, not calls but read parts are filtered out. For the VAF-lees filter, a VAF distribution is regarded as a beta-mixture distribution and component beta distributions are identified. A component of which the peak corresponds to a low VAF is then considered as the error distribution. The details of all filters are in Additional file 2: Text S1.

Figure S2.

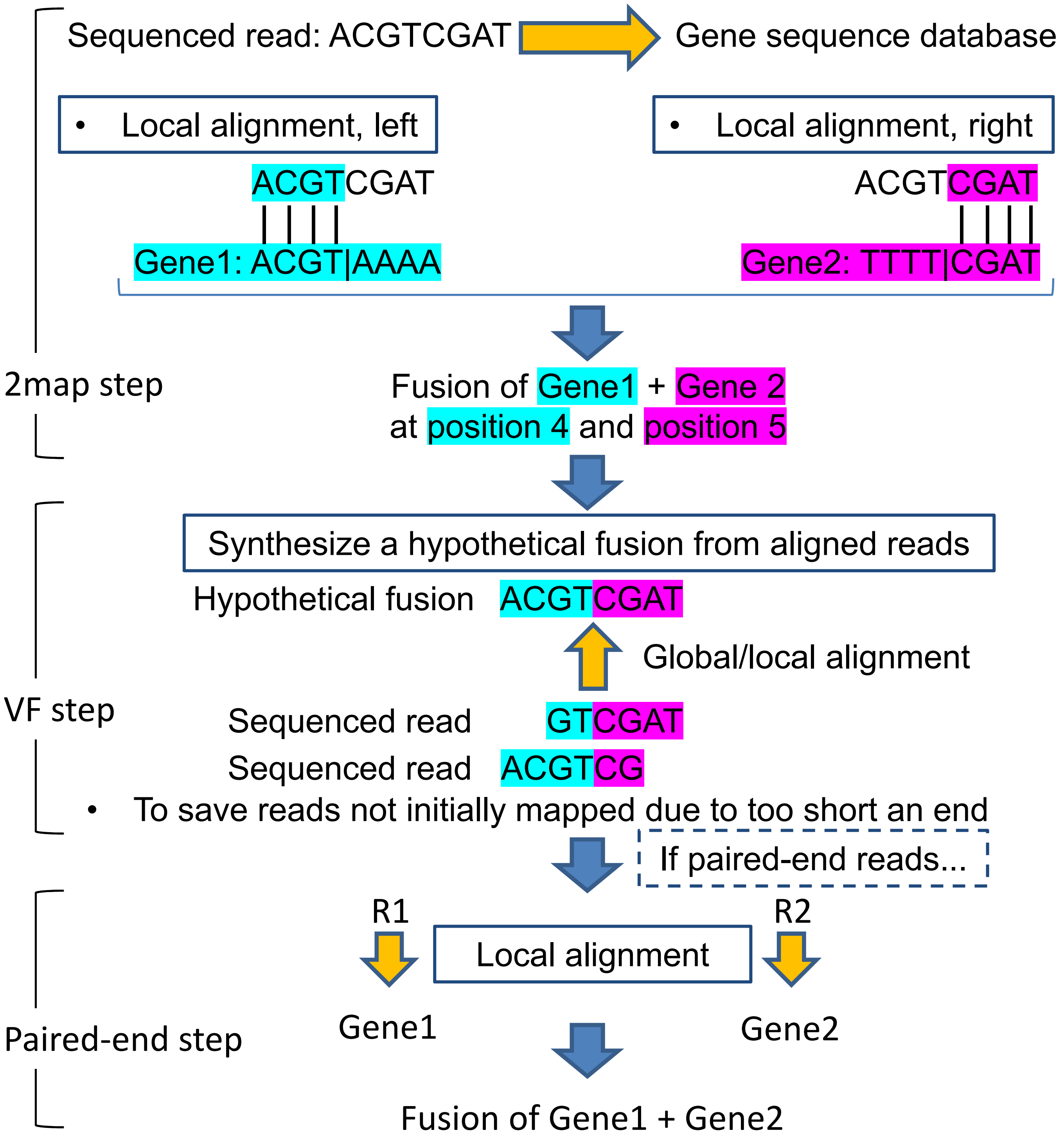

Figure S2. Principle of our fusion detection method.
At the 2map step, we utilize local alignment for facile extraction of breakpoint positions. At the VF step, we generate a virtual (hypothetical) fusion and align reads to it to save reads that are not mapped in 2map call as one read end is too short to be aligned. At the paired-end step, we map each (R1 and R2) of the paired-end sequenced reads separately.

Figure S3.

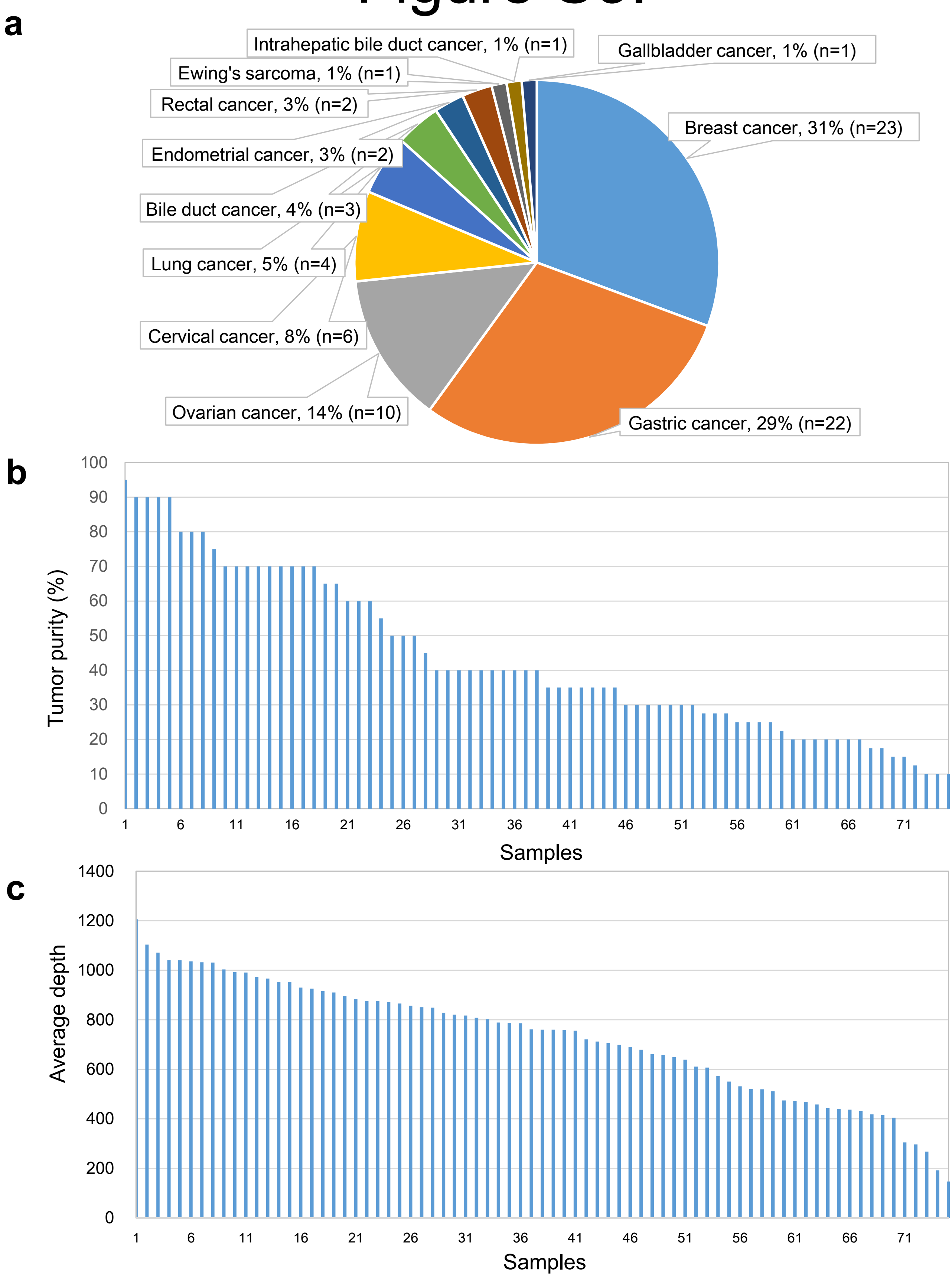

Figure S3. Descriptive statistics of the 75 FFPE samples.
a Cancer type. **b** Histologically determined tumor purity. **c** Average read depth.

Figure S4.

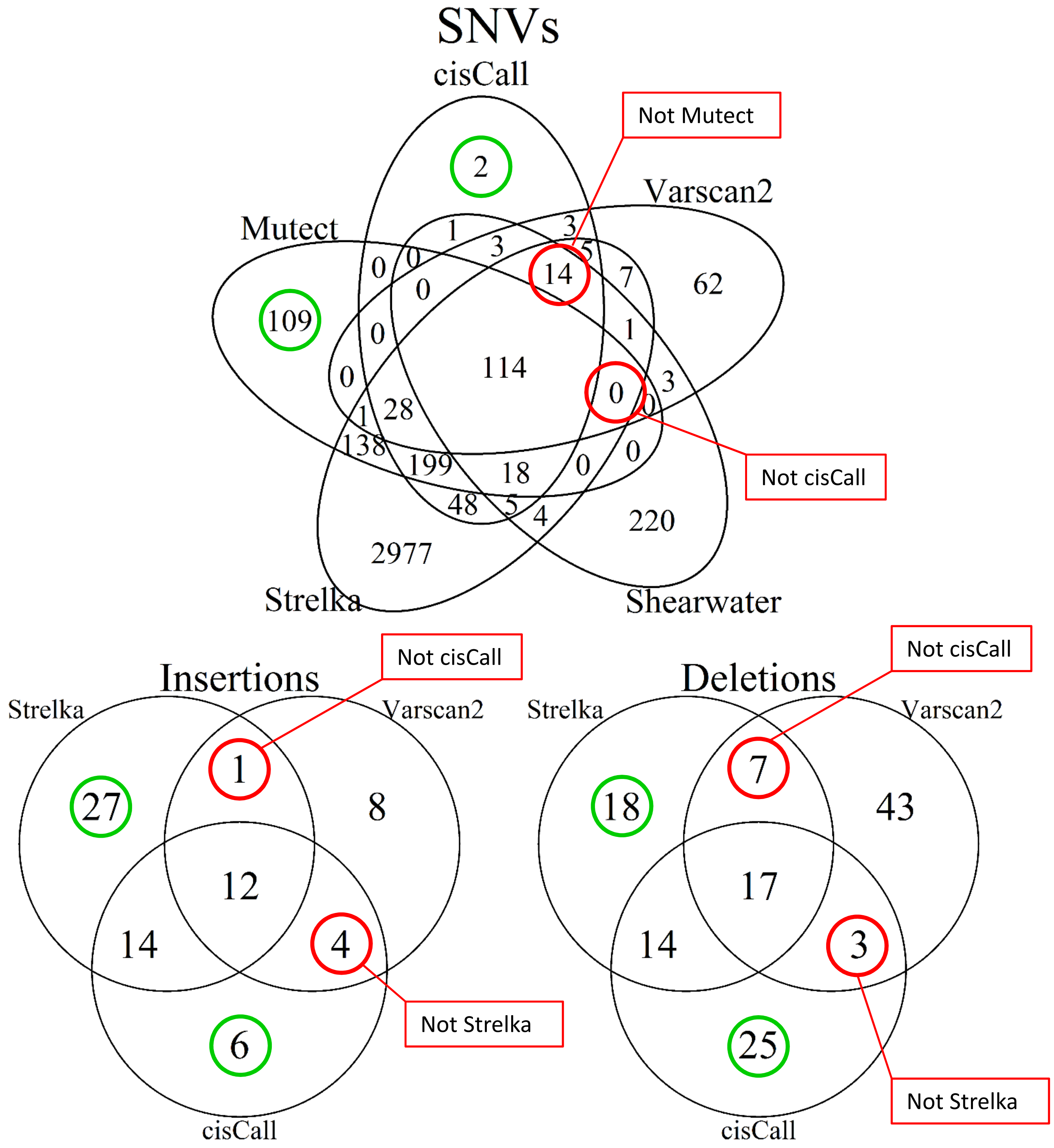

Figure S4. Venn diagram of SNVs and indels called from FFPE sample data using various calling tools.
We excluded known germline variants using SNP databases and possible germline variants by filtering calls with VAFs of 40–60% and ≥ 96% because we did not use matched normal samples. The subsets of isolated calls for mass spectrometry validation are indicated in green circles for calls made only by the tool and in red for calls *not* made by the tool only. We randomly selected calls from the subsets for the validation experiment and only some of the probes were successfully designed.

Figure S5.

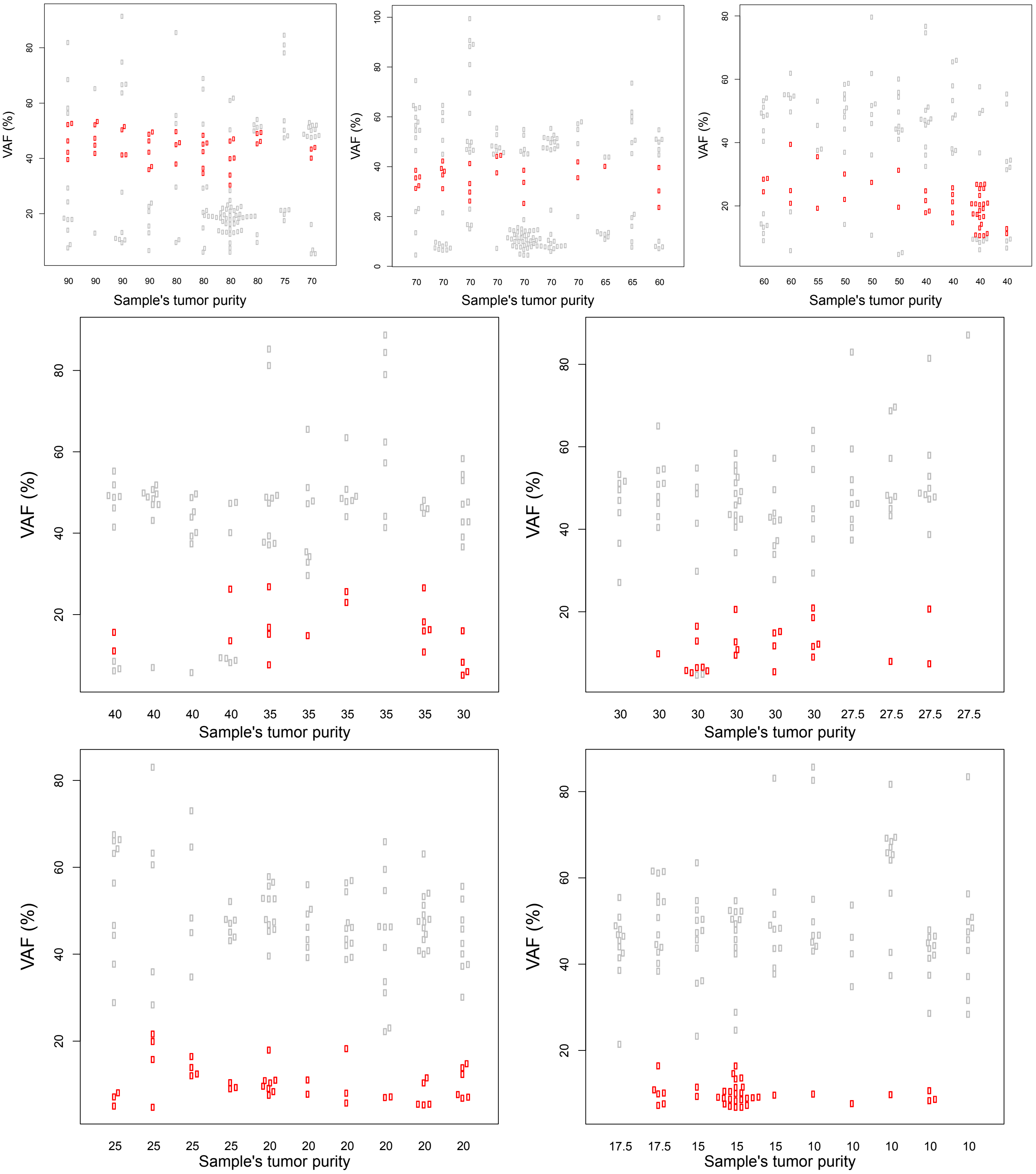

Figure S5. Beeswarm plots of tumor purity versus the VAFs of SNVs/indels called from the FFPE samples.

Samples are ordered by tumor purity, which was histologically determined by pathologists. Red circles correspond to calls with VAFs around (± 10%) half of the tumor purity. Note that the calls may include personal germline SNPs because we did not use matched normal samples in the variant calling. VAFs may also easily be influenced by germline CNVs and somatic CNAs.

Figure S6.

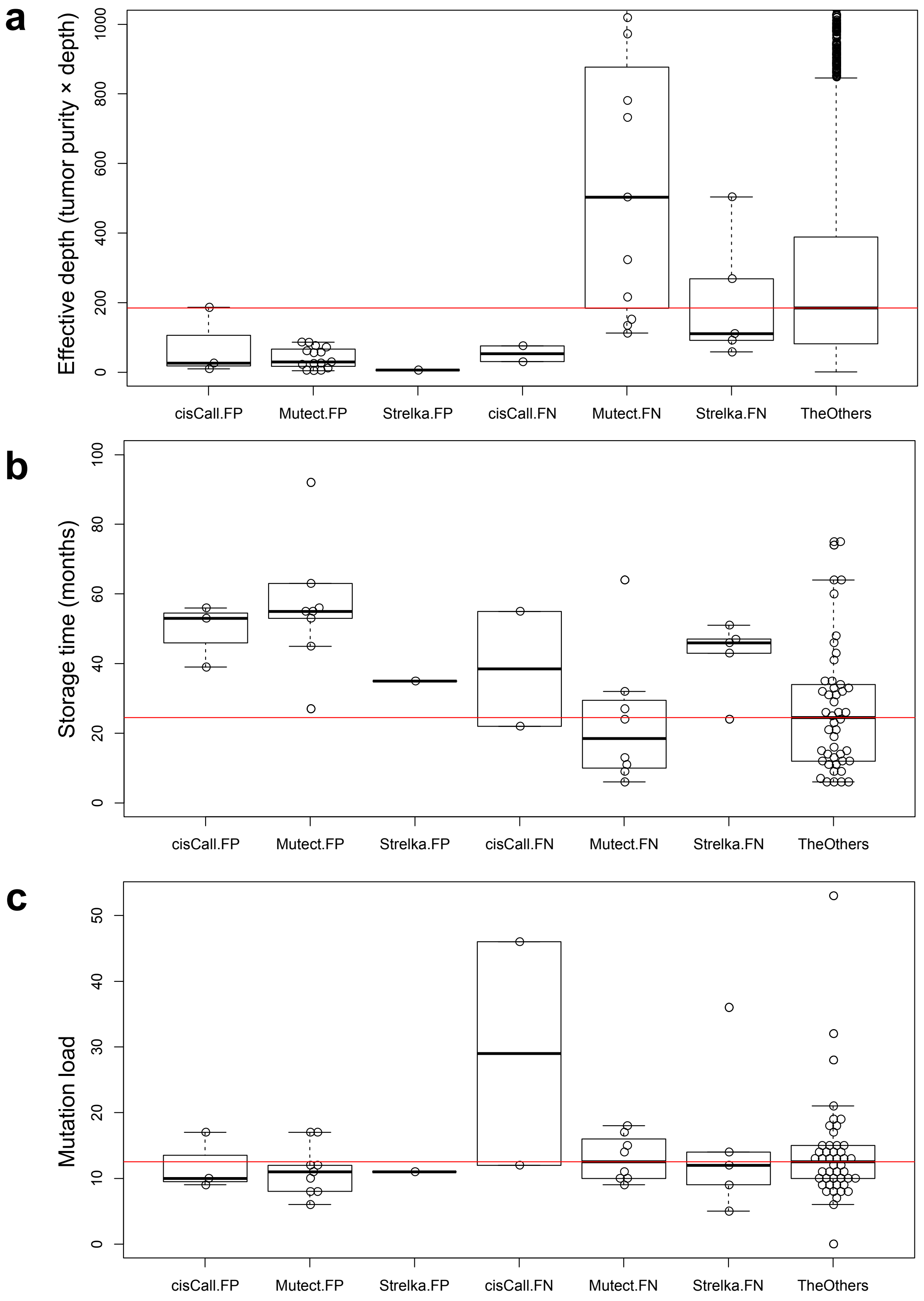

Figure S6. SC-FP and SC-FN variants stratified by the indicated factors.
FP and FN on the *x*-axis represent SC-FP and SC-FN, respectively. **a** Stratified by the effective depth (tumor purity × the depth of each variant). The points represent variants. **b** Stratified by FFPE storage time. We do not show one sample with an exceptionally long storage time (182 months). **c** Stratified by mutation load per 477 kb target size.

Figure S7. (part1)

Amplifications

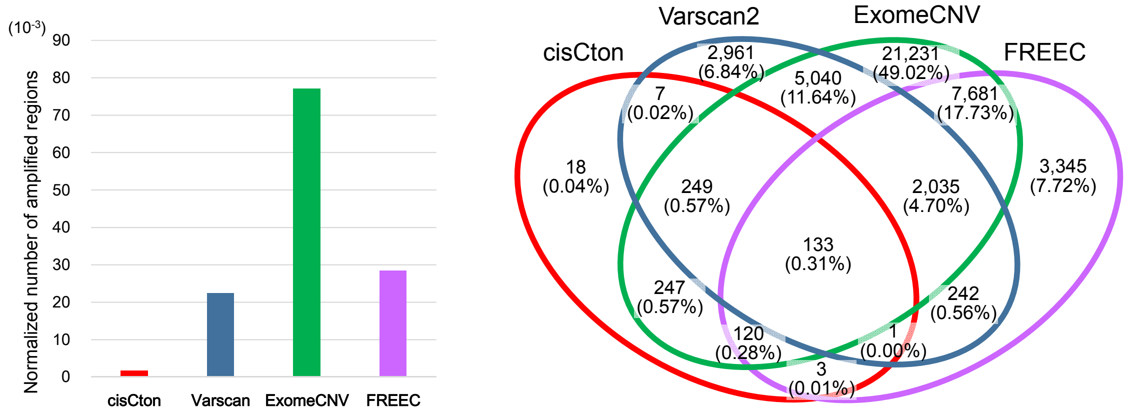

Number of amplified regions

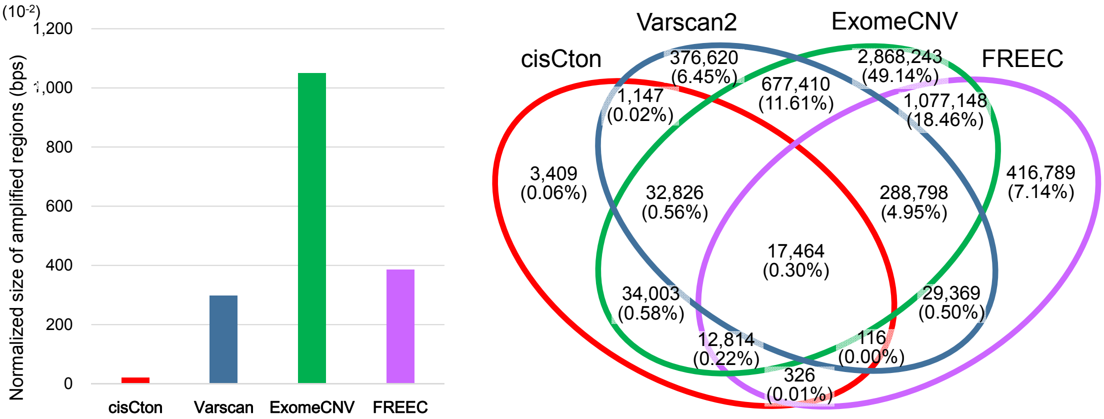

Size of amplified regions

Figure S7. Venn diagram of CNAs called from FFPE sample data using various calling tools. Number and total size of regions called as amplified and deleted out of the target capture regions (left), and the Venn diagram (right). We excluded long regions for fusion capture. The normalized numbers and sizes were based on target region size (477 k bps).

Figure S7. (part2)

Deletions

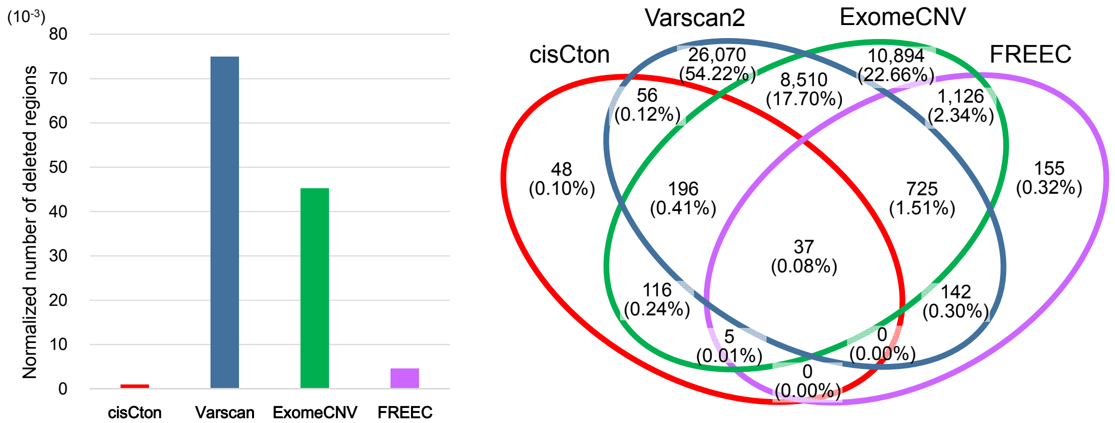

Number of deleted regions

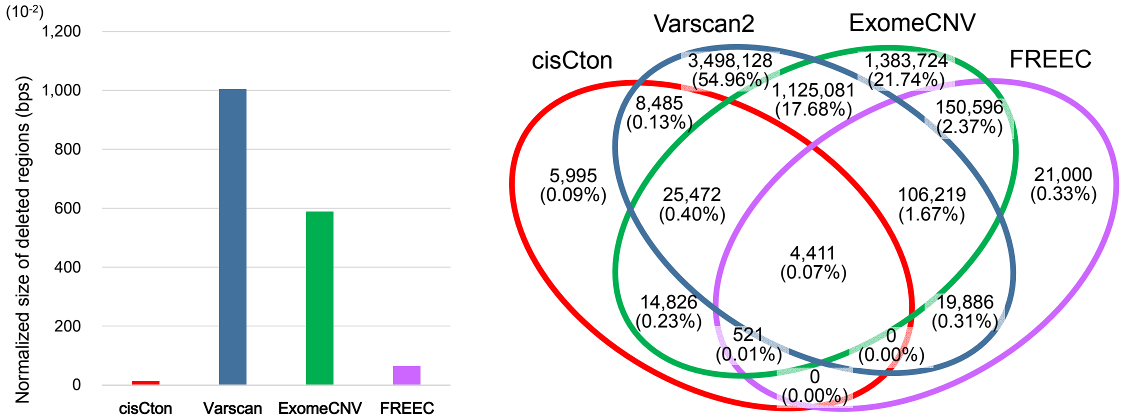

Size of deleted regions

Figure S8.

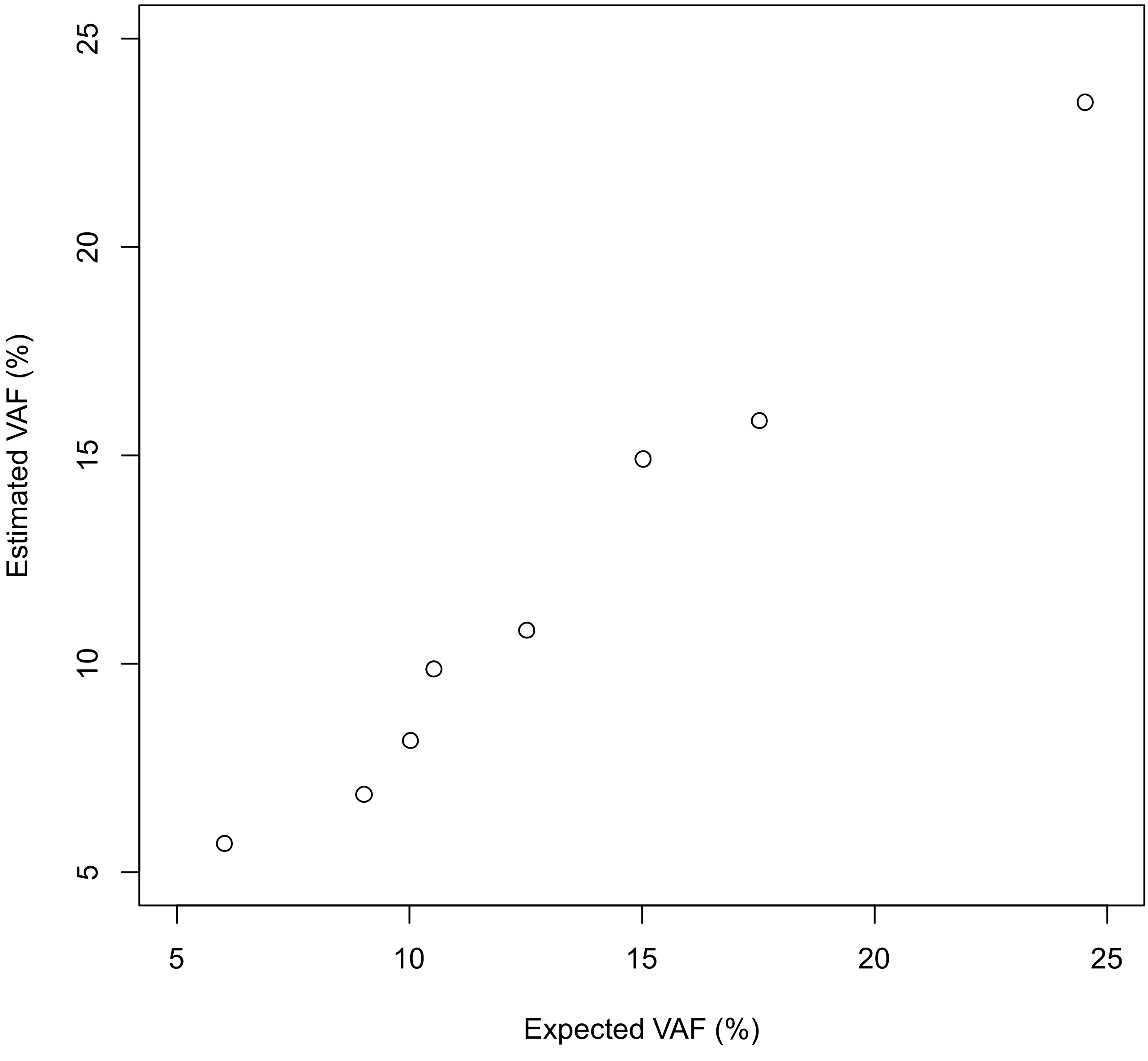

Figure S8. Expected and estimated VAFs for SNVs inserted in commercially available FFPE standard material.

The values of the expected VAFs were provided by the manufacturer, and the estimated VAFs were computed by cisMuton.

Figure S9.

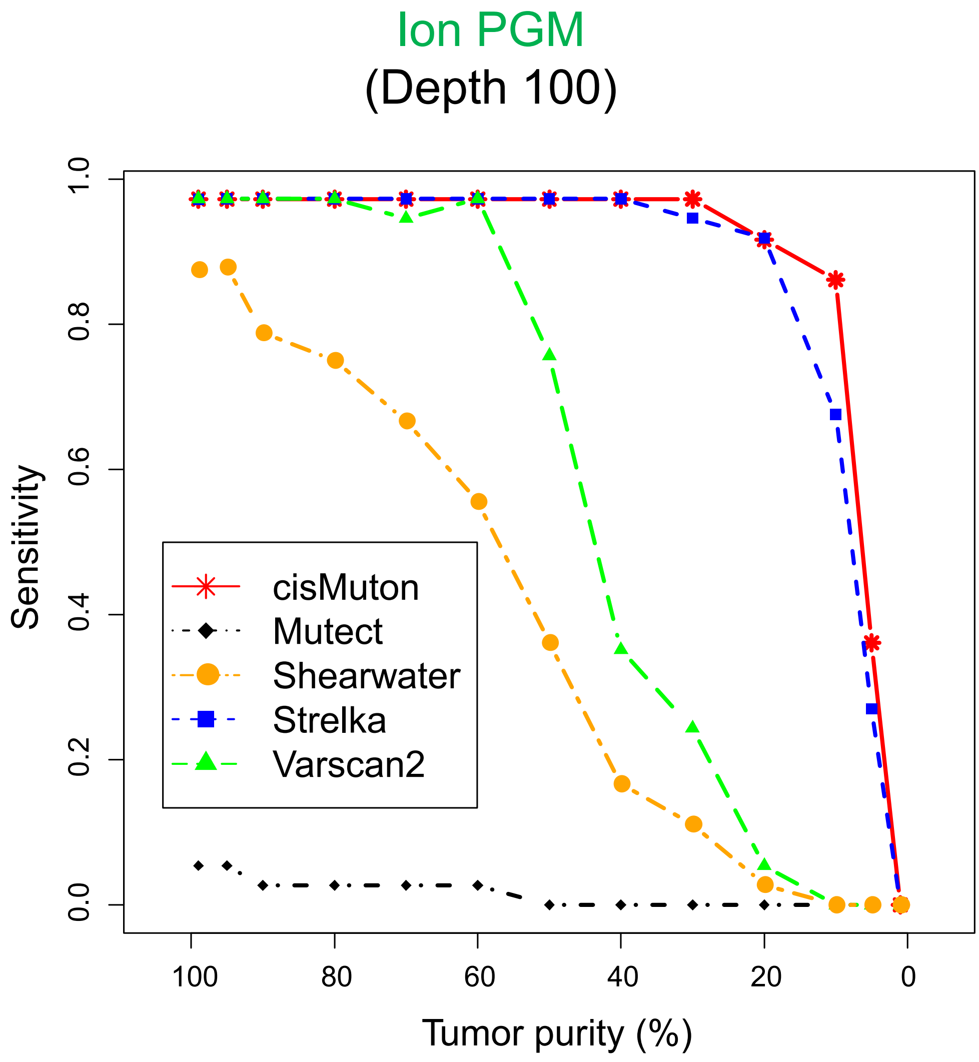

Figure S9. Sensitivity estimation based on semi-simulated Ion sequencing data. The simulation settings are the same as in Fig. 1b.
